# Invasive Fungal Disease: From Diagnostic and Therapeutic Failure to Integrated Care

**DOI:** 10.3390/jof12100727

**Published:** 2026-09-29

**Authors:** Pilar Rivas-Pinedo, José Millán Oñate Gutiérrez

**Affiliations:** 1 Medical and Diagnostic Mycology Group, Department of Microbiology, Faculty of Medicine, Universidad Nacional de Colombia, Bogotá 111321, Colombia; 2 One Health Research Group for Infectious Diseases, Keralty, Department of Internal Medicine, Clínica Colsanitas S.A., Clínica Sebastián de Belalcázar, Cali 760045, Colombia; 3 Clinical Research and Education Group (GIEDCO), Clínica de Occidente, Cali 760035, Colombia

## 1. Introduction

Invasive fungal diseases remain an important cause of severe illness and death worldwide, and timely recognition and effective treatment are still difficult to achieve [1]. Diagnostic options have expanded substantially through fungal biomarkers, molecular assays, mass spectrometry, rapid diagnostic platforms, and antifungal susceptibility testing. Antifungal therapy has also evolved. Even so, access to better tools does not necessarily translate into earlier diagnosis or a favorable clinical response. Outcomes depend on a sequence of decisions that begins with recognizing the patient at risk and continues through appropriate sampling, test selection and interpretation, organism identification, antifungal treatment, adequate drug exposure, source control, and host recovery (Contribution 1, 2).

This sequence is also useful for reading the contributions to this Special Issue. Diagnostic failure may arise from delayed clinical suspicion, inadequate sampling, limitations of individual assays, incomplete organism identification, or failure to act on diagnostic information in time (Contribution 1). An unfavorable therapeutic response, in turn, should not automatically be attributed to microbiological resistance. Inadequate antifungal exposure, diagnostic uncertainty, persistent infection foci, high fungal burden, host-related factors, and pathogen characteristics may all contribute to apparent or true treatment failure (Contribution 2). The difficulty, therefore, lies not only in choosing a test or an antifungal agent but also in connecting diagnostic and therapeutic decisions throughout the course of care.

This Special Issue was conceived to bring together clinical, diagnostic, epidemiological, therapeutic, and translational perspectives on invasive fungal disease across different pathogens, host populations, and healthcare settings. The contributions collected here illustrate both the progress made and the persistent gap between the availability of diagnostic or therapeutic advances and their effective integration into patient care.

Several contributions address this problem from the diagnostic perspective. In aspergillosis, the performance and interpretation of biomarkers vary with the host population, clinical form, specimen type, and pre-test probability (Contribution 3). Their usefulness consequently depends on how and when they are incorporated into the diagnostic workup. This is particularly relevant in critically ill patients. In a cohort of patients with severe COVID-19, Blaize et al. found that estimates of invasive pulmonary mold infection differed according to the classification system applied, and fulfillment of criteria for probable COVID-19-associated pulmonary aspergillosis was also influenced by the intensity of diagnostic sampling (Contribution 4). Case definitions and sampling practices can therefore affect both epidemiological estimates and interpretation of clinical outcomes.

Uncommon organisms pose a different challenge. The report of *Candida viswanathii* candidemia associated with contaminated milrinone described failure of a rapid blood-culture molecular panel to identify the yeast. Extended microbiological identification, reconstruction of medication exposure, and lot traceability later helped link the infection to a broader iatrogenic event (Contribution 5). Beyond the rarity of the species, the case shows why broad mycological identification capacity remains important and why microbiology may need to be connected with pharmacovigilance and healthcare surveillance.

## 2. Epidemiology, Surveillance, and Local Context

Fungal epidemiology is neither static nor geographically uniform. Global recommendations provide an essential framework, but their application is influenced by local species distribution, susceptibility patterns, patient populations, and healthcare practices.

In Colombia, an interrupted time-series analysis of 1294 microbiologically confirmed cases of invasive candidiasis between 2019 and 2024 identified persistent seasonality and temporal fluctuations, including an increase followed by a decline, without statistically significant changes in level or slope after the predefined pandemic interruption point (Contribution 6). A simple pre- versus post-pandemic interpretation would therefore be insufficient. Longitudinal surveillance offers a better way to distinguish sustained epidemiological patterns from transient variation.

A post-pandemic candidemia cohort from a high-complexity Colombian teaching hospital provided a more local view. Non-*albicans Candida* species predominated, and fluconazole resistance was identified in 28.6% of *Candida parapsilosis* isolates (Contribution 7). Mortality remained high. Species-level identification and antifungal susceptibility testing are clinically relevant in this setting because they can influence drug selection and institutional treatment policies. The multicenter surveillance study and the institution-specific cohort therefore provide different but complementary perspectives on candidiasis epidemiology (Contribution 6, 7). Contemporary guidance similarly emphasizes accurate species identification, susceptibility testing when clinically relevant, and consideration of local epidemiology in therapeutic decision-making [2].

Regional heterogeneity was also apparent in the study of infant–juvenile paracoccidioidomycosis in Argentina. During the 15-year study period, the northwest and northeast endemic regions showed different temporal and clinical patterns (Contribution 8). Cases in the northwest occurred more consistently over time, whereas the northeast showed a more episodic pattern, with differences in organ involvement between regions. Endemic areas should therefore not automatically be treated as epidemiologically homogeneous. Geographic exposure remains part of the diagnostic reasoning for endemic mycoses [3].

## 3. Critical Illness and Host-Dependent Decision Making

Critically ill patients show particularly clearly why uniform approaches to invasive fungal disease can be difficult to apply. The IFUCISTRATEGY survey, which included specialists from 51 hospitals in Spain, found broad agreement on the role of bronchoalveolar lavage galactomannan in suspected pulmonary aspergillosis. Practice was more variable, however, for empirical treatment, suspected azole resistance, and management of antifungal exposure during extracorporeal membrane oxygenation or renal replacement therapy (Contribution 9). These areas remain unsettled despite substantial specialist experience.

The multicenter cryptococcosis cohort from Türkiye adds another perspective (Contribution 10). One-year mortality was 46.7%. Altered consciousness and disseminated infection were independently associated with increased mortality, whereas higher serum albumin was associated with improved survival; age also remained independently associated with mortality (Contribution 10). Central nervous system involvement was more frequent among people living with human immunodeficiency virus (HIV), but mortality was comparable between HIV and non-HIV groups. Risk assessment in cryptococcosis therefore extends beyond HIV status alone, in keeping with contemporary guidelines that recognize the heterogeneity of disease across host populations and clinical presentations [4].

Host recovery is equally important in selected immunocompromised patients. In a pediatric cohort of ten patients with proven or probable invasive mold disease who underwent allogeneic hematopoietic stem cell transplantation after clinical improvement, no relapse of invasive mold disease or infection-related mortality was observed through day +100 (Contribution 11). The retrospective design and small sample limit generalization. Still, the findings suggest that complete clinical or radiological resolution may not always be required before transplantation in carefully selected children. The degree of infection control has to be weighed against the urgency of the underlying indication for transplantation.

Standardized recommendations remain important, but these studies show that their interpretation depends on the host, local epidemiology, antifungal exposure, and the clinical consequences of delaying other necessary interventions.

## 4. Emerging Pathogens, Resistance, and Healthcare-Associated Risk

Emerging fungal pathogens create additional difficulties because they may fall outside routine identification systems, challenge established susceptibility assumptions, and complicate infection-prevention practice.

A nosocomial cluster of *Trichosporon asahii* in a Colombian high-complexity hospital involved nine patients, including five healthcare-associated infections and four colonization events (Contribution 12). Environmental cultures did not microbiologically confirm a reservoir, although irregularities in ventilation-system maintenance were considered a plausible epidemiological contributor. That distinction should be preserved. Infection-control action may be necessary before a source is definitively established, but epidemiological plausibility is not equivalent to microbiological proof.

The *T. asahii* cluster and the *C. viswanathii* event also expand the meaning of fungal surveillance (Contribution 5). Surveillance is not limited to tracking common invasive pathogens. It also requires recognition of unexpected species, detection of clustering, reliable species-level identification, and communication among clinical microbiology, infection prevention, pharmacovigilance, and other institutional safety systems.

Local resistance data are another part of that surveillance. Fluconazole resistance among *C. parapsilosis* isolates in the Colombian candidemia cohort shows how susceptibility patterns may affect institutional antifungal strategies (Contribution 7). Resistance, together with the limited number of established antifungal classes, also keeps therapeutic innovation clinically relevant [5].

One contribution explored this problem at a preclinical level. A nanoemulsion-delivered chromium(III)–triazole complex evaluated against fluconazole-resistant *Candida albicans* showed antifungal activity in vitro and reduced renal fungal burden in a murine model of invasive candidiasis (Contribution 13). These findings do not establish clinical efficacy, but they provide a basis for further preclinical investigation.

## 5. Integrating Diagnosis, Treatment, and Stewardship

Diagnostic and therapeutic failure often converge in the same clinical course. Delayed or uncertain diagnosis can lead to inappropriate treatment; incomplete species identification may obscure clinically relevant susceptibility patterns; inadequate antifungal exposure can mimic microbiological resistance; and uncontrolled infection foci or host-related factors may prevent response despite selection of an active antifungal agent (Contribution 1, 2).

Antifungal stewardship provides a practical framework for connecting these elements. Diagnostic stewardship, appropriate use and interpretation of susceptibility testing, optimization of antifungal exposure, source control, and structured reassessment of non-response should be viewed as parts of the same clinical process rather than as isolated interventions (Contribution 1, 2). Systematic-review evidence suggests that antifungal stewardship can reduce antifungal consumption and improve selected process or economic outcomes, although interventions and outcome measures remain heterogeneous [6].

Making these elements work together requires coordination among clinical teams, microbiology laboratories, pharmacists, infection-prevention professionals, and other specialists caring for high-risk patients. The need for such coordination appears repeatedly across this collection, from biomarker interpretation and candidemia surveillance to unusual healthcare-associated infections and transplantation following recent invasive mold disease (Contribution 3–7, 11, 12). A diagnostic or therapeutic advance is useful only if it can be translated into an appropriate and timely clinical decision.

## 6. Remaining Gaps and Priorities for Research

The Special Issue includes contributions from Colombia, Argentina, Türkiye, Spain, Italy, and France (Contribution 4–13). Latin American studies, in particular, should not be viewed solely through a framework of resource limitation. They contribute longitudinal surveillance data, antifungal resistance information, outbreak investigation, pediatric transplant experience, epidemiology of an endemic mycosis, conceptual work on diagnostic and therapeutic failure, and preclinical therapeutic research (Contribution 1–2, 6–8, 11–13).

Several gaps remain. Prospective implementation studies are scarce. Pharmacokinetic/pharmacodynamic optimization, therapeutic drug monitoring, cost-effectiveness, access to diagnostics and antifungals, and external validation of diagnostic and stewardship pathways are still incompletely addressed. Disease-specific evidence for mucormycosis and other difficult-to-treat mold infections is also limited within this collection.

Future work should therefore examine not only whether a diagnostic method is more sensitive or whether a new antifungal compound has activity, but how these advances perform once introduced into heterogeneous clinical settings. Relevant questions include how quickly diagnostic results alter treatment, when susceptibility testing changes management or outcomes, how antifungal exposure can be optimized in complex hosts, and which stewardship strategies remain effective across different healthcare systems.

## 7. Conclusions

The persistent challenges of invasive fungal disease cannot be explained by diagnostic sensitivity, antifungal resistance, or drug availability alone. Across different pathogens, hosts, and healthcare settings, the contributions to this Special Issue point to the importance of interpreting diagnostic and therapeutic advances within their clinical and epidemiological context.

Further progress will require better tests and treatments, but also better integration of epidemiological surveillance, clinical mycology, antifungal therapy, source control, host factors, and multidisciplinary reassessment in routine care. The next step is not only to improve individual diagnostic and therapeutic tools but also to make them work together more consistently across different patients, pathogens, and healthcare settings.

## Data Availability

No new data were created or analyzed in this study. Data sharing is not applicable to this article.
